# Bacterial response to Ti-35Nb-7Zr-5Ta alloy incorporated with calcium, phosphate and magnesium

**DOI:** 10.1007/s10856-023-06717-3

**Published:** 2023-04-28

**Authors:** Bárbara Araújo dos Reis, Natalia Da Ponte Leguizamón, Yumi Chokyu Del Rey, Leandro Fernandes, Cássio do Nascimento, Luis Geraldo Vaz

**Affiliations:** 1 Department of Diagnosis and Surgery, School of Dentistry, São Paulo State University (UNESP), Araraquara, 14800900 Brazil; 2 Department of Dental Materials and Prosthodontics, School of Dentistry, University of São Paulo (USP), Ribeirão Preto, 14049-900 Brazil; 3 Department of Dental Material and Prosthodontics, School of Dentistry, São Paulo State University (UNESP), Araraquara, 14800900 Brazil

## Abstract

High implant survival rates have been achieved in recent decades due to continual modifications in implant design and surface topography, however there is still an ongoing quest to control peri-implant bone loss. The objective of this work was to develop Ti-35Nb-7Zr-5Ta (TNZT) alloys, perform physicochemical and morphological characterization of their surface modified by electrolytic oxidative plasma technique with ions related to osseointegration and lastly evaluate bacterial colonization in vitro. Three groups were evaluated: C group (polished TNZT), CaP group (sodium β glycerophosphate + calcium acetate) and Mg group (magnesium acetate). Before and after anodizing the surfaces, physicochemical and morphological analyses were performed: scanning electron microscopy with field emission gun (FEG-SEM), energy dispersion spectroscopy (EDS), X-ray diffraction (DRX), wettability (goniometer) and roughness (rugometer). Controlled and treated specimens were contaminated with unstimulated saliva collected from 10 healthy volunteers. Then, biofilm samples were collected and up to 35 microbial species, including commensal and pathogenic microorganisms, were identified and quantified by the *Checkerboard DNA-DNA Hybridization* method. The CaP group modified the surface morphology in the form of pores, while the Mg group modified it in the form of flakes. The contact angle was significantly smaller in the CaP group. The average roughness was higher in the CaP and Mg groups. A smaller total amount of bacteria was identified in the Mg group and relevant differences were found in the microbial profile associated with different surface treatments. Therefore, considering the microbiological profile and for the prevention of peri-implantitis, the Mg group presented more satisfactory and encouraging results for the manufacture of dental implants

**Graphical Abstract:**

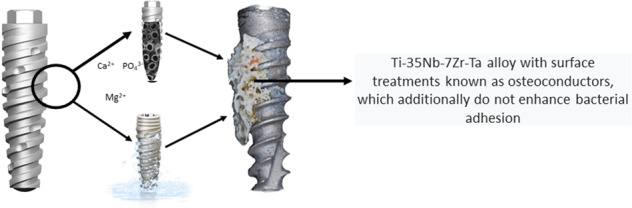

The constant evolution of dental implants has established implant dentistry as one of the best rehabilitation treatment plans, with survival rates above 90% in recent years [1], compared to 66% or less in the first years of technology [2]. However, this data is far from ideal, especially in patients at high risk of complications such as diabetics, periodontal patients, and patients using bisphosphonates, among others, which justifies the constant search for the development of new biomaterials.

The main improvements in the performance of implants are directed towards making titanium (Ti) alloys and improving their surface. The passive film of Ti is predominantly composed of TiO_2_ [3]. Ti alloys, on the other hand, can have a varied composition of oxides, depending on the alloying elements present, which can modify the protective property of the Ti oxide. For example, it is known that Nb leads to the formation of Nb_2_O_5_, Zr, ZrO_2_, Ta, Ta_2_O_5_, V, VO_2_ and Al, Al_2_O_3_. And, that the Ti oxide modified by the oxides of Nb, Zr and Ta present better stability and, therefore, can provide better protection than the oxides of Al and V [3, 4]. Niobium (Nb) is a typical β stabilizer of Ti alloys and has many advantages for having the same cubic centered body structure as titanium (ccc), similar atomic radius, without presenting biological toxicity. Previous works [5, 6] showed that the addition of Nb and Ta elements to Ti alloys can considerably decrease the modulus of elasticity. Zr suppresses the omega (ω) phase in quaternary alloys and is also considered responsible for the decrease in the modulus of elasticity reaching values of 55 GPa when these alloys are compared to binary alloys containing similar amounts of Nb [6–8]. The improvement of surfaces is carried out by various techniques, such as acid etching, hydrothermal treatment, sandblasting, laser, plasma electrolytic oxidation (PEO), and anodizing, among others. PEO is capable of modifying the titanium oxide film layer using high voltages, making it more adherent and porous, with better biological properties [9], in addition to enabling the incorporation of ions into the oxide layer.

The incorporation of calcium (Ca^2+^) and phosphate (PO_4_^3−^) ions is commonly performed by PEO. It is known that incorporation of these ions helps the formation of bone apatite and greater adhesion and proliferation of osteoblasts [10]. Beneficial effects are also reported with the incorporation of magnesium ions (Mg^2+^) in implants to significantly increasing the adhesion of osteoblastic cells through signaling pathways mediated by a5b1 and b1 integrins [11]. Despite this encouraging data, it is necessary to assess the interference of these innovations in future clinical complications, both related to material properties and their impact on microbial biofilm formation. Changing surfaces can influence the adhesion and proliferation of bacteria and consequently, result in marginal bone loss around implants. When an implant surface is exposed to microorganisms, there will be adhesion or repulsion of bacteria depending on the result of different interactive forces, these interactions being highly influenced by surface wettability, surface charge (positive or negative electromagnetic interactions), roughness and other chemical characteristics of the surface [12].

It is believed that divalent cations such as Mg^2+^ and Ca^2+^ can affect the initial fixation of bacteria in biofilm formation, bridging the gap between molecules due to the attraction of cations and the extracellular matrix of negatively charged bacteria, which plays a positive role in structural stability of the biofilm [13]. However, the influence of divalent cations on the physiological functions of bacterial biofilms is reflected by the electrolyte composition, such as concentration and type of cation as well as the bacterial species.

Regarding the proportions of bacterial species, surface roughness seems to be an important factor in the colonization capacity of certain species [14]. Bacteria preferentially adhere to surfaces that conform to their size, as this maximizes the bacteria’s surface area. Surface irregularities proportional to the size of the bacteria increase its contact area and, therefore, the binding potential, while irregularities much larger than the bacteria size approach the binding potential of a flat surface [15]. For a given surface, different species of bacteria adhere differently, as they have different physicochemical characteristics. Generally, bacteria with hydrophobic properties prefer surfaces of hydrophobic material; those with hydrophilic characteristics prefer hydrophilic surfaces [12].

The crystallinity of the oxide formed on the surface also seems to influence the bacterial response to the biomaterial. When titanium dioxide is crystallized in the anatase phase, antibacterial properties are expressed through the formation of anionic oxygen species [16].

Factors such as chemical composition, hydrophilicity, crystalline phase, nanotopography and surface charges can also be modified on titanium or titanium alloy surfaces to favor or disprove antibacterial activity. Strategies such as the development of new alloys and the functionalization of implant surfaces must be explored to obtain a biomaterial capable of optimizing osseointegration without increasing the potential for bacterial adhesion, and in an even better situation, being able to inhibit bacterial adhesion and prevent infections.

Titanium β alloys and surface treatments with CaP and Mg have proven osteogenic potential, but little is known about their influence on bacterial colonization, making it essential to evaluate the potential of these new implant surfaces in controlling biofilm. If biomaterials used as dental implants increase the osteoinductive/osteoconductive/ostegenic potential and also increase the adhesion of bacteria, it will be necessary to carry out a careful analysis to assess the risk/benefit of such therapies. Hence, it is expected that surfaces developed to enhance osseointegration do not cause an imbalance in the oral microbiota, or, in an ideal situation, that they are able to reduce colonization by species considered pathogenic. Therefore, the aim of this study was to develop a titanium β alloy composed of Ti-35Nb-7Zr-5Ta and characterize the experimental surfaces by the PEO technique with incorporation of Ca^2+^, PO_4_^2−^ and Mg^2+^ ions. Morphological, chemical, wettability and surface roughness changes were evaluated, as well as the effect of surface treatment on the colonization of up to 35 species of microorganisms present in the oral cavity.

## Material and method

The Ti-35Nb-7Zr-5Ta alloy was obtained by the arc-voltaic casting method, with inert atmosphere, controlled by vacuum pump and argon flow. After casting, the ingots were subjected to a heat treatment of homogenization at 1000 °C for 8 h (vacuum), then were forged and machined in the form of discs [4]. All discs were submitted to finishing and polishing in a polish machine with silicon carbide-SiC sandpaper (Norton Abrasivos do Brasil, São Paulo, SP, Brazil) of 120, 320, 600, 1200 and 2000 grains.

The specimens were mounted in an electrochemical cell connected to a digital multimeter and an energy source (N5771A, Agilent Technologies do Brasil São Paulo, SP, Brazil) to carry out electrolytic oxidation by plasma. Group C is composed of untreated discs. For the CaP group, we used a protocol for the formation of micropores with incorporation of Ca^2+^ and PO_4_^3−^, in an electrolyte composed of 0.04 mol/L of sodium β-glycerophosphate (Sigma-Aldrich Co, St Loius, Mo, USA) plus 0 .35 mol/L calcium acetate (Sigma-Aldrich Co, St Loius, Mo, USA). The current source was adjusted to 300 V with varying electrical current intensity, starting at 2.5 A, for 60 s. For the Mg group, we used a magnesium acetate electrolyte at a concentration of 0.1 mol/L [4] with a voltage of 200 V, current of 2 A for 60 s.

The topography of the samples was evaluated before and after PEO in a scanning electron microscope (Jeol JSM6610LV, Akishima, Tokyo, Japan) to evaluate the surface morphology. For semi-quantitative chemical analysis, we used the energy dispersive X-ray (EDS) technique (INCA 250, Oxford Instruments, Concorde, New Hampshire, USA), in order to detect the elements present on the surface. X-ray diffraction (XRD) with copper Kα was used to evaluate the crystal structure of all samples. The constants of each phase were calculated from low angle peaks with known Miller indices, cell type and internal standard [17].

The wettability of the samples was evaluated by the value of the contact angle (AC) using a goniometer (Ramé-Hart 100-00, Succasunna, NJ, USA), with drops of distilled water dispensed on the surface of the samples by a syringe always the same size of droplet. This AC was measured after 20 s and repeated three times for each sample. Linear surface roughness (Ra) and area roughness (Sa) were measured before and after the anodizing procedures, in a roughness meter (Mitutoyo SJ 400 – Mitutoyo Corporation - Japan). Three measurements were taken on each surface and the mean between readings was determined as the value of Ra (µm) and Sa of each sample.

For analysis and evaluation of the results obtained, mean and standard deviation were used. The results of Ra and Acs exhibited normal distribution and were analyzed using two-way analysis of variance, *two-way* ANOVA followed by Tukey’s test (*α* = 0.05).

Unstimulated human saliva was used as a contaminating medium for disc incubation. Exclusion criteria was under 18 years old, pregnancy, lactation, smoking, presence of systemic diseases, active caries lesions, periodontal disease and use of antibiotics in the last 3 months or medications that could influence the periodontal condition. Participants were instructed in writing and orally about the objectives of the study and the steps involved in their participation. The study was approved by the Ethics Committee of the School of Dentistry of Araraquara, registration number 46325221.0.0000.5416. Five millilitres of unstimulated saliva was collected from 10 periodontal healthy individuals aged between 27 and 35 years old(mean age 31.40 ± 1.14) and mixed in a 50 mL Falcon tube. Five discs from each group were incubated in 2 mL eppendorfs, containing 1 mL of the contaminating medium. This volume was enough to cover the discs preventing leakage of contaminant medium. After seven days of incubation at 37 °C, the discs were removed from the microtubes and the biofilm formed on the specimens was collected individually with the aid of a microbrush brush rubbed over the surface of the disc for 20 s. The tip of each microbrush was placed in identified microtubes containing 250 µL of TE buffer solution to preserve the stability of the genetic material of the biofilm samples. Then, 150 µL of 0.5 M sodium hydroxide (NaOH) solution was added to the microtubes in order to allow cell lysis and suspension of genomic DNA of microorganisms in the solution. The samples were kept at room temperature until the time of microbial assays to avoid freeze-thaw cycles that could affect the integrity of the DNA. All contamination tests were carried out under aseptic conditions, in a laminar flow hood and using sterile instruments and gloves.

Thirty-three bacteria and 2 Candida species were defined as target species, described in Table 1. The genomic DNA of all target species was purchased from the American Type Culture Collection (ATCC^®^, USA). The genomic probes of the 35 target-detection species were made according to the protocol established by the genomic marker manufacturer (AlkPhos Direct Labeling and Detect System, GE Healthcare, Buckinghamshire, UK).

**Table 1:** Microbial species (and respective ATCC number) used for preparation of the target-specific DNA probes

	ATCC number
Bactérias	
*Aggregatibacter actinomycetemcomitans* a	29523
*Bacteroides fragilis*	25285
*Capnocytophaga gingivalis*	33624
*Campylobacter rectus*	33238
*Campylobacter gracilis*	33236
*Escherichia coli*	2193
*Enterococcus faecalis*	23834
*Fusobacterium nucleatum*	51299
*Klebsiella pneumoniae*	33693
*Lactobacilos casei*	334
*Lactobacillus acidophilus*	4356
*Pseudomonas aeruginosa*	27853
*Peptostreptococcus anaerobius*	27337
*Porphyromonas endodontalis*	35406
*Porphyromonas gingivalis*	33277
*Prevotella intermedia*	25611
*Prevotella melaninogenica*	25845
*Parvimonas micra*	33270
*Prevotella nigrescens*	25261
*Pseudomonas putida*	12633
*Staphylococcus aureus*	25923
*Streptocuccus gordonii*	10558
Streptococcus mitis	49456
*Solobacterium moorei*	CCUG39336
*Streptococcus mutans*	25175
*Streptococcus parasanguinis*	15911
*Streptococcus salivarius*	25975
*Streptococcus sanguinis*	10556
*Streptococcus pneumoniae*	55143
*Streptococcus gallolyticcus*	700066
*Treponema denticola*	35405
*Tanerella forsythia*	43037
*Veillonella parvula*	10790
Candida	
*Candida dubliniensis*	44508
*Candida tropicalis*	66029

After collecting the biofilm from the discs, samples were evaluated by *Checkerboard DNA-DNA Hybridization* technique, a semi-quantitative molecular method of microbiological diagnosis that is based on the occurrence of hybridization reactions (DNA-DNA binding) between labeled DNA probes by fluorescence for each of the target species for detection and the genomic DNA of the microorganisms present in the analyzed samples, as described in [18]. Briefly, standards containing a known number of microbial cells (10^5^ and 10^6^ cells) for each of the target species were analyzed simultaneously with the analyzed biofilm samples to serve as a comparison standard for quantification. Thus, by detecting the intensity of fluorescent signals generated by the DNA-DNA hybridization reaction and comparing them to controls, it is possible to estimate the number of cells of each target species in the investigated biofilm samples. ANOVA-Type (ATS) and Wald-Type (WTS) were used to determine the interaction effects of surface treatments and target species on the microbial counts. Generalized Estimating Equations (GEE) modeling was performed for multiple comparisons between groups. Statistical significance was set at *p* < 0.05 level. All statistical analyses were performed using R Statistical Software (Version 4.0.0, R Foundation for Statistical Computing, Vienna, Austria).

## Results

**Fig. 1:**
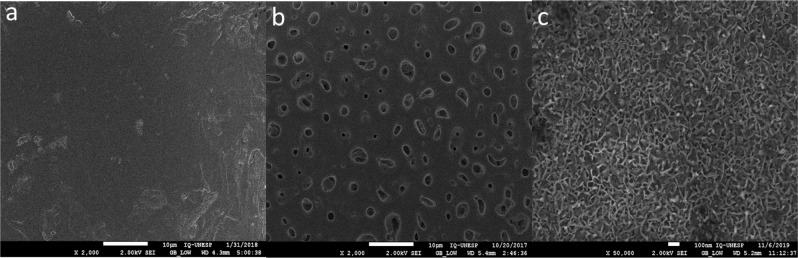
Microscopy of study surfaces. **a** C group; **b** CaP group; **c** Mg group

The C group presented a smooth surface with grooves compatible with the sandpaper used for polishing (Fig. 1a), without formation of specific structures. The CaP group resulted in the formation of uniform surfaces with pore characteristics (Fig. 1b) and the Mg group resulted in the formation of uniform surfaces with flaky characteristics (Fig. 1c).

**Fig. 2:**
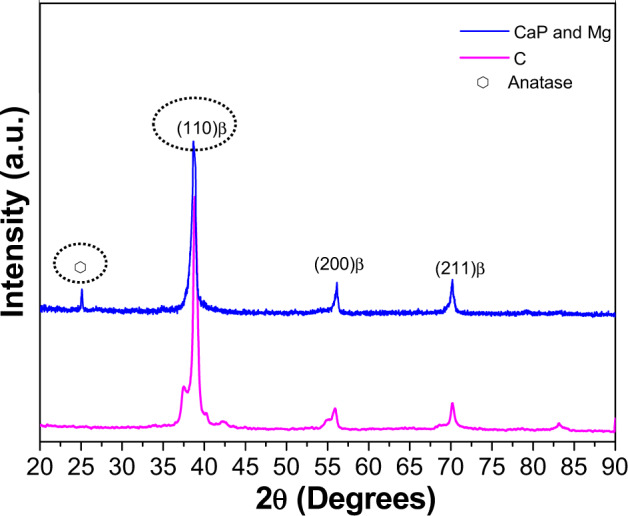
XRD analysis of C, CaP and Mg groups with a predominance of the β phase and presence of anatase in CaP and Mg groups

X-ray diffraction (XRD) patterns of treated and untreated surfaces at an incidence angle of 10° to 90° are shown in Fig. 2. The untreated TNZT surface (pink line in Fig. 2) exhibited only the β phase, as shown by the presence of peaks (110), (200) and (211). No alpha phase was detected. This is due to the presence of beta-stabilizing elements (Nb, Zr and Ta) in the material that suppresses the formation of the alpha phase. After the heat treatment, the peak 2θ = 25.5° appeared in the treated groups, indicating the presence of the anatase phase.

**Fig. 3:**
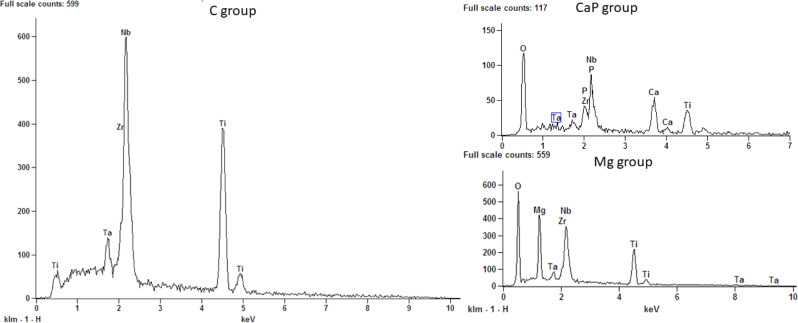
EDS of study samples

EDS analysis showed incorporation of Ca^2+^ and PO_4_^3−^ in the CaP group and Mg^2+^ in the oxide layer of the Mg group, and did not show contamination after the anodizing process (Fig. 3).

Significant differences were obtained as a function of anodizing (*p* < 0.0001). The mean roughness of the samples from the treated groups was statistically higher than that of group C, with no statistical difference between CaP and Mg groups (Table 2). In general, the mean square values (Sz) are in agreement with the mean roughness values, demonstrating the good homogeneity of the samples. Contact angle measurements showed that all groups present values below 90◦ using distilled water, thus suggesting a qualitatively hydrophilic character (*θ* > 90°). There was a statistically significant difference in the values of CAs in the Mg group compared to CaP and C groups (Mg = 41.4°, CaP = 50.4°, C = 56.6°). (Table 2).

**Table 2:** Linear surface roughness (Ra), area roughness (Rz), wettability (CA) of the study groups

Group	Ra (µm)	Sz (µm)	Ca (°)
C	0.26 (±0.3)	2.30 ± 0.4	55.6 (±2.9)
CaP	0.54 (±0.8)	4.09 ± 0.4	50.4 (±4.5)
Mg	0.61 (±0.3)	4.66 ± 0.3	41.4 (±3.1)

**Fig. 4:**
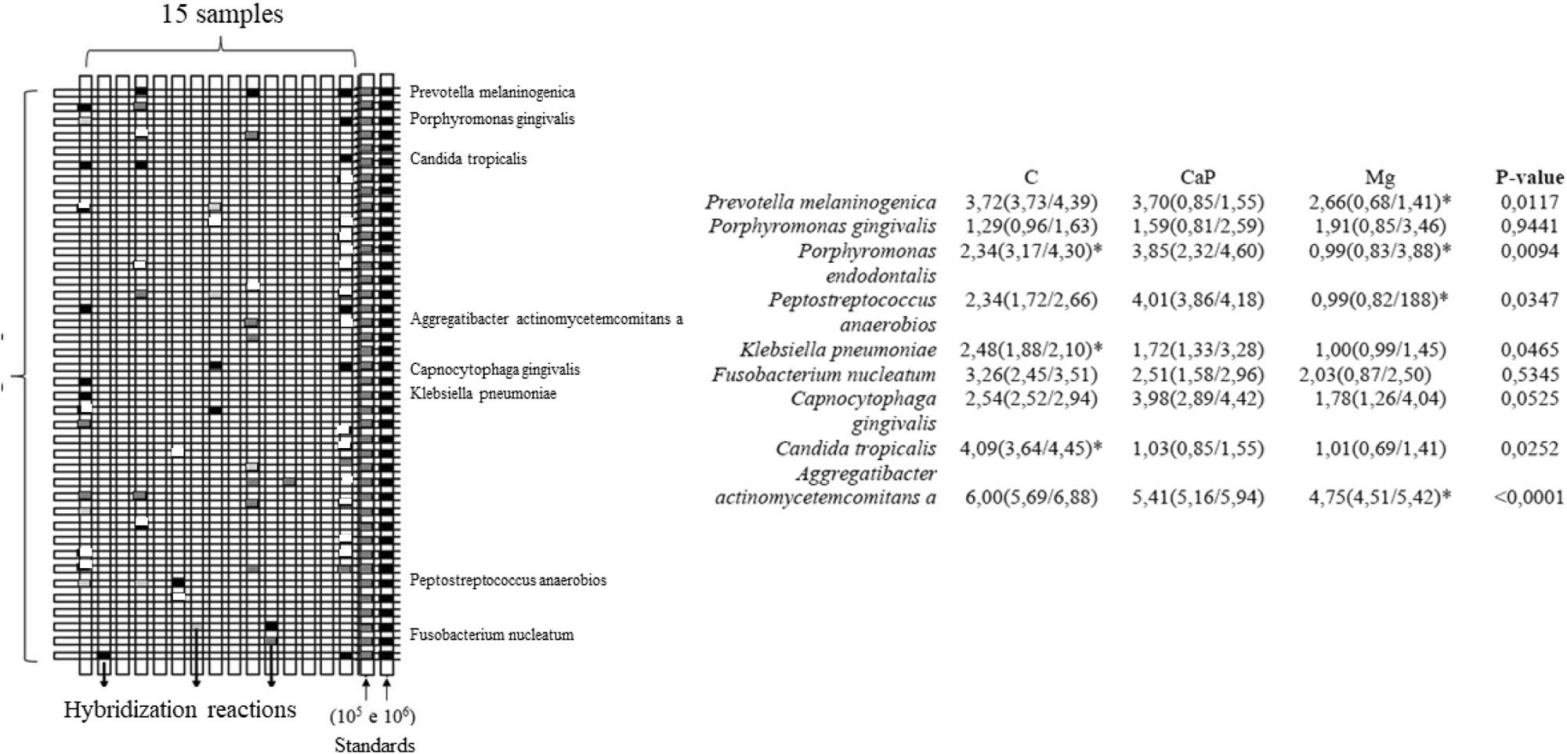
Schematic drawing of membrane after revealing the hybridization signals and medians (first/third quartiles) of microbial counts (×10^5^) obtained by DNA-DNA Checkerboard hybridization technique and the respective *P* values for comparison between groups obtained by GEE analysis with a significance level of 0.05

Among the 35 microorganisms investigated, 9 were detected in all groups, described in Fig. 4, which illustrates the median, first quartile, third quartile, maximum and minimum values of the total microbial count recorded for the 3 study groups.

Non-parametric Brunner and Langer test (Non-parametric analysis of longitudinal data in factorial experiments) was used to analyze the inference of treatments on the total microorganism count, without discriminating between the different investigated species. The relative effect of the group factor (treatment) was significant for the microorganism count, according to Wald-Type Statistics (WTS): *p* < 4.23 × 10^−5^ and ANOVA-Type Statisticis (ATS): *p* < 4.36 × 10^−6^. The analysis of multiple comparisons (pairwise multiple comparisons), with Bonferroni correction, showed differences in the total bacterial count between groups C and Mg (*p* < 5.13 × 10^−5^). Species and treatment factors showed significant isolated effects, *p* = 0.0000 and *p* < 1.15 × 10^−13^, respectively. Treatment/species interaction also had a significant effect; *p* < 1.18 × 10^−6^. GEE (Generalized Estimating Equations) test was used to investigate each of the species in the different proposed treatments (Fig. 4).

The formation of hybridization signals at different intensities is observed at the intersection formed between the investigated samples and the standards with the probes of the 35 detection target species. The intensity of the generated signal is directly proportional to the number of cells of the target species present. The strict anaerobic bacteria *Prevotella melaninogenica*, *Porphyromonas endodontalis* and *Peptostreptococcus anaerobios*, and the green complex bacteria *Aggregatibacter actinomnycetemcomitans* were identified in smaller amounts in the Mg group. The opportunistic species *Klebsiella pneumoniae* and *Candida tropicalis* showed preferential colonization in group C. As for the red and orange complex bacteria, *Porphyromonas gingivalis* and *Fusobacterium nucleatum* respectively, no clear trend was identified between groups and all groups had relatively low counts of such species.

## Discussion

Factors such as biomaterial chemical composition, hydrophilicity, surface crystalline phase, nanotopography and surface charges can be modified on surfaces or titanium alloy to provide osteoblastic and/or antibacterial activity. Previous studies have shown that titanium concentrates more biofilm mass and greater amounts of microorganisms [19]. In the present study, we used TNZT titanium alloy β and anodizing technique to modify the surface with the addition of ions with osteogenic potential, and we evaluated the bacterial response using the checkerboard technique on these surfaces.

Our results agree with previous studies that showed that the incorporation of ions into the TNZT alloy may not enhance biofilm formation depending on the concentration and time of exposure to the ions [13, 20, 21]. Therefore, if titanium surfaces are attractive to bacteria, strategies such as chemical modification and functionalization of implant surfaces must be altered in order to inhibit adhesion and prevent infection.

Due to their excellent biocompatibility and good mechanical properties, titanium β alloys are promising materials for making implants [5]. These alloys have superior properties over the Ti-6Al-4V alloy that is used clinically, such as lower Young’s modulus and better biocompatibility. Furthermore, the TNZT alloy is known for its extremely high corrosion resistance and thermodynamic stability [22]. Corrosion resistance can reduce the chance of early implant failure caused by the combined effect of corrosion and wear. With the rapid development of surface modification technologies over the last few years, many surface treatment techniques have been reported to improve the wear resistance of titanium alloys, as reported by Oliveira et al. [23] which obtained an excellent tribocorrosive behavior on surfaces modified with Ca, P and Mg.

The incorporation of Ca, P and Mg ions is described as capable of altering characteristics in titanium and its alloys [13, 20], and in this work there was a modification of roughness, hydrophilicity, surface crystalline phase, chemical composition and surface charges, probably responsible for the change in bacterial adhesion pattern. Divalent cations are related to increased biofilm formation, especially due to their ability to bind to the surface of negatively charged bacteria [13], so special attention is needed when incorporating these ions into implant surfaces to verify the influence of the modification on bacterial behavior. Calcium participates in bacterial fixation mediated by adhesin, being able to modify the adhesin structure, and when it binds to the Ca^2+^ structure, it strengthens and facilitates the extension of the cell surface protein to the substrate [20]. However, researchers have observed a protein-mediated inhibitory effect caused by the addition of Ca in *Staphylococcus aureu*s biofilms [21].

*P. aeruginosa* strain was increased with the addition of Mg^2+^, but this was not observed with the other three strains of *P. aeruginosa* tested [24]. However, other authors found that Mg degradation is responsible for increasing the alkalinity of the environment, pH above 9, and consequently inhibiting bacterial survival. Robinson et al. [25] considered that the characteristic of Mg degradation in a physiological solution could result in rapid increases both in the concentration of Mg^2+^ and in the pH value in the solution, the latter being responsible for the antibacterial function of Mg. This is because bacteria can generally be alive in an environment with a pH range of 6.0–8.0, in which bacteria can maintain a cytoplasmic pH compatible with the ideal functional and structural integrity of cytoplasmic proteins [26]. Consequently, acidic and alkaline environments are not suitable for bacterial survival. Probably this characteristic is responsible for a lower expression of strict anaerobic bacteria *P. melaninogenica, P. endodontalis* and *P. anaerobios*, and *A. actinomnycetemcomitans*.

The treated groups (CaP and Mg), still had a lower expression of opportunistic species like *K. pneumoniae* and *C. tropicalis*. These results corroborate the justification that ions may not enhance biofilm formation depending on the concentration and time of exposure to the ions [13]. Furthermore, wettability characteristics and crystalline phase of the oxide may have influenced this result. A correlation can be attributed among the chemical composition of the oxide, the formation of the anatase phase and wettability with surface coating by biofilm, since the group treated with Mg^2+^, presented the anatase phase and a smaller contact angle, and had lower expression of some bacterial species identified in this study. Hydrophilic surfaces are highly desirable in implant dentistry because they make it difficult for bacteria to adhere to the surface and, consequently, reduce biofilm formation [14], in addition to optimizing osseointegration. The total microbial count was lower in the Mg group, which is probably due to a higher surface wettability and the ability of Mg^2+^ to change the environment to alkaline, making it difficult for bacteria to survive.

It is known that bacterial fixation and aggregation are influenced by calcium and magnesium. Care must be taken to analyze the other factors that interfere in the process such as, cation concentration and specificity, surface hydrophobicity, roughness, and crystalline phase, among others. Some authors suggest that the average roughness and chemical composition influence the surface wettability [12]. In our work, the TNZT alloy by itself is already hydrophilic as it presents a contact angle smaller than 90°, but its contact angles were even smaller with the proposed treatments, and its roughness was considerably higher.

The Ra and Sz values of the CaP and Mg groups showed statistically higher values than those of the C group. The other test groups were considered minimally rough (Ra 0.5–1 µm) and the C group was considered smooth (<0.5 µm) [27]. Even on minimally rough surfaces, there are reports of a positive correlation between increased minimal roughness and osseointegration, as demonstrated by Park et al. [28] They compared turned implants to three different groups of oxidized implants. Turned implants had a Ra of 0.54 µm, oxidized implants in group 1 had an Ra of 0.68 µm, oxidized group 2 had a Ra of 0.80 µm, and oxidized implants in group 3 had a Ra of 0.88 µm. After 6 weeks in rabbit bone, group 3 oxidized implants demonstrated significantly more bone-to-implant contact and higher removal torque than the other groups of implants. Sul et al. [29, 30] evaluated electrolytes that changed the chemical composition and topography containing P, S, Ca and Mg.

Chemically modified implants demonstrated greater removal torque and more bone-implant contact than controls, especially surfaces reinforced with Ca and Mg, despite the Ra/Sa values being similar to the control group. This suggests that minimal roughness can interfere with biological processes, and theoretically rougher surfaces, while having a larger area available for bone-implant contact, also have a larger area available for microbial adhesion. However, it is not possible to evaluate a single parameter separatedly, all surface characteristics that may influence these processes must be evaluated, and the bacteria assessed in the study is probably more sensitive to surface chemistry than to roughness.

Oliveira et al. [23] observed that chemical modification of surfaces with the addition of Mg in the electrolyte with CaP increased the level of rutile in the oxide layer, probably due to an increase in the conductivity of the electrolyte. In this work, as the processes were performed separately, the conductivity was probably similar and only the anatase phase was expressed in both groups. The anatase phase is known for characteristics that can improve the hydroxyapatite growth process [31]. Recent studies have pointed to a reduction in biofilm formation on anatase surfaces [12, 32], but the exact mechanism related to these results is not known.

Therefore, the role of cations in biofilm formation is multifactorial. It is believed that the presence of calcium and magnesium affects the initial fixation of bacteria. They bridge molecules, modify cell surface adhesins and reduce apparent surface charge and surface potential. In contrast, the role of divalent cations in the physiological functions of bacterial biofilms is reflected by electrolyte composition, such as cation concentration and cation type, as well as bacterial species and strains. Therefore, if titanium surfaces are attractive to bacteria (surface charge, roughness, crystalline phase and hydrophobicity), strategies such as functionalization of implant surfaces must be changed in order to inhibit adhesion and prevent infection.

This study, however, does not simulate the insertion of implants in the recipient bed, where there are specific conditions such as friction between the implant and the bone, and the presence of hundreds of microorganisms present in the oral environment. Additional research validating the capacity of two treatment protocols developed in coating dental implants, according to the degree of attachment to the substrate and the influence of two treatments on the process of microbial colonization and osseointegration can help to understand the relationship between implants and the development of biofilms, and the success and long-term implants survival.

## Conclusion

Despite the limitations of this study, based on the results obtained, it is possible to conclude that:

In addition to Nb, Zr and Ta, the ligand in the β phase stabilized;

MAO with sodium β-glycerophosphate and calcium acetate was able to promote micropores on the surface of the TNZT bond, and electrolyte with Mg promoted a surface with a flock appearance;

Roughness of TNZT bond was altered to minimally rough with surface modification by MAO with CaP and Mg groups;

The CaP and Mg groups show lower contact angle values, being considered more hydrophilic than the treated ligament;

A total microbial counting was significantly lower in Mg group;

The microbial profile was different for the groups investigated with less expression of *P. melaninogenica*, *P. endodontalis*, *anaerobic Peptostreptococcus* and *A. actinomnycetemcomitans* in Mg group.

For microbiological evaluation, the Mg group shows more satisfactory and encouraging results for the preparation of dental implants.
